# Ultrasonographic findings after Achilles tenotomy during Ponseti treatment for clubfeet: Is ultrasound a reliable tool to assess tendon healing?

**DOI:** 10.1007/s11832-014-0610-3

**Published:** 2014-09-30

**Authors:** P. Nasr, L. Berman, A. Rehm

**Affiliations:** Addenbrookes University Hospital NHS Trust, Cambridge, UK

**Keywords:** Clubfoot, Ponseti, Achilles tenotomy, Tendon ultrasound, Tendon healing

## Abstract

**Introduction:**

Several studies have claimed ultrasound to be useful and accurate in assessing the healing phase of Achilles tendons after tenotomy during Ponseti treatment for clubfoot deformity. The purpose of our study was to assess the healing process of Achilles tendons ultrasonographically after tenotomy as part of Ponseti clubfoot management and to assess the effects of previously not considered ultrasound properties (anisotropy, partial volume effect), and whether these practical considerations affect accurate measurements which have been claimed possible in previous studies.

**Materials and methods:**

We monitored the post-tenotomy healing process in 15 patients (22 tendons) using high frequency ultrasound for a minimum of six months (range 6–14 months). The scanning was discontinued once a tendon looked normal or when the appearance remained unchanged between scans. We also studied nine patients (11 tendons) who had undergone Achilles tenotomies up to seven years previously (range 34–83 months).

**Results:**

In the immediate postoperative period, ultrasound showed large variations in the distance of the tenotomy from the calcaneum as well as the obliquity and completeness of the surgical division. We encountered pitfalls in the use of ultrasound to define healing stages that were not described previously. Sonography was inaccurate and subjective in assessing both completeness of the surgical division and tendon measurements. Despite ultrasonographically proven incomplete tendon division in 63 % of cases, the clinical effect of an immediate increase of passive foot dorsiflexion from the pretenotomy position with an obvious palpable tendon gap was achieved in all patients. At the end of the study, 65 % of tendons did not achieve a normal appearance.

**Conclusions:**

We do not think that routine ultrasound studies are of any value as an adjunct to clinical assessment intra- and post-operatively. It can give misleading information regarding the need to complete the tenotomy, which may increase risks associated with a further pass of the scalpel blade.

## Introduction

Idiopathic congenital talipes equinovarus is a common condition affecting between 0.6 and 6.8 per 1,000 live births [1]. The Ponseti technique is well recognised in the management of clubfoot deformity with high success rates [2]. This technique has decreased the need for extensive corrective surgery [3]. Following the serial application of casts as per Ponseti’s original descriptions [4, 5], a percutaneous Achilles tenotomy is undertaken to enable or improve foot dorsiflexion. The natural history of healing of the tenotomised tendon is not well understood. Several studies have claimed ultrasound to be useful and accurate in assessing the healing phase [6, 7]. In three previous studies, the reported length of time for tendons to achieve continuity based on ultrasound images and clinical assessment varied between six weeks to 45 months [6–8].

We monitored the post-tenotomy healing process using high frequency ultrasound to understand the appearances of the healing tendon and the timescale over which the tenotomy healed. We also studied patients that had undergone an Achilles tenotomy several years previously to ascertain the longer-term effect of the Ponseti treatment. Furthermore, we studied patients without clubfoot deformity to use their tendons as controls.

## Materials and methods

Approval was obtained from our local Ethics Committee. The approval number was 09/H0308/143.

We performed ultrasound studies on three groups of children. Only idiopathic clubfeet were included. Syndromic feet were excluded.

### Group 1

We prospectively studied 22 Achilles tenotomies in 15 patients who underwent serial clinical and sonographic evaluation of the tendoachilles after tenotomy and the tendon of the normal foot of patients with unilateral clubfoot.

In all new presentations, surgery was performed between two and 11 months of age. One revision procedure was performed at six months; the original surgery having been performed at another hospital when the child was two months of age.

### Group 2

A further group of 11 Achilles tenotomies in nine older patients (seven unilateral and two bilateral) were scanned at ages ranging from three-years-old to seven-years-old at several years following the procedure. They had undergone surgery between the ages of two and 5.5 months.

Neither group included syndromic clubfeet.

### Group 3

A control group was comprised of seven children with 14 normal feet who were undergoing ultrasound for unrelated pathology such as urinary tract infections.

#### Surgical procedure

Tenotomies were performed percutaneously in the operating room under general anaesthetic using a size 15 blade (Swann Morton, Sheffield, UK). The tendon was palpated proximal to the calcaneal insertion, and the blade was inserted on the medial and anterior border of the Achilles tendon cutting the tendon towards its lateral and posterior aspect. This resulted in an immediate passive dorsiflexion of the feet from the starting position with an obvious palpable gap in the Achilles tendon, suggesting complete division. After tenotomy, 5 ml of 0.25 % Chirocaine was injected locally. Steri Strips, Primapore dressing and an above the knee Plaster of Paris cast were applied. No surgical or anaesthetic complications were encountered, and all patients were discharged on the day of surgery. In seven patients (11 clubfeet) with severe plantar flexion, we performed serial casting after tenotomy to gain more dorsiflexion to facilitate boot and bar application. We aimed for 20° of dorsiflexion. Four patients had one cast change, two had two changes, and one had four changes. The mean postoperative casting period was 21 (17–31) days for all patients. None of the patients required a further Achilles tenotomy. In two of these patients, the post-tenotomy Pirani score was 0, but the toes were not perfused within the first intraoperative cast with the foot in maximum dorsiflexion. Therefore, we reapplied the cast in the operating theatre in a position where the toes were perfused with the foot in less dorsiflexion.

#### Ultrasound follow-up

During clinical follow up, the children underwent ultrasonographic evaluation of their tenotomy. We aimed to scan patients at regular intervals, starting at four weeks after the procedure to ascertain whether we could observe similar appearances as in previous studies [6, 8–10]. We then aimed to scan patients at their routine physiotherapy boots and bar checks at eight, 12, and 18 weeks after surgery. Scanning intervals, however, did vary between patients. Not all parents would have agreed to come for research ultrasound had they not coincided with the physiotherapy follow-up. Scans were performed in longitudinal and transverse scanning planes using a Toshiba Aplio XG apparatus (Toshiba Medical Systems, Crawley, UK) with an 18 MHz matrix linear array transducer. All scans were performed by a single operator (LB), who is a Radiology Consultant experienced in paediatric and musculoskeletal ultrasound. All patients were scanned in the prone position with the feet in a neutral position.

Ultrasound observations included the completeness of division, obliquity of the tenotomy, and the length of the gap between the severed ends of the tendon. We assessed the dynamic integrity of the tendon during passive ankle dorsiflexion and plantarflexion on serial scans. The evolution of the healing process was documented, noting a change in the echogenicity from amorphous bridging tissue, which, nevertheless, appeared to result in functional continuity, to the characteristic striated echopattern of normal tendons. We attempted to measure tendon gaps, but, due to the very subjective nature of the measurements in such small tendons and the tendon ends being irregular and ill defined, we concluded that any measurements were too inaccurate to subject to statistical analysis. These clinically and ultrasonographically united tendons were compared with the normal control patients. We initially scanned some patients prior and directly after the tenotomy, but as Mangat et al. [7] previously reported, we also did not think that this contributed to the study of healing.

## Results

### Results for group 1 patients (22 tendons in 15 patients)

All initial studies were performed within four weeks of surgery (range 0–27 days; mean 17.8 days). All group 1 patients (Tables 1, 2) were followed up with scans for a minimum of six months (range 6–14 months) apart from one whose tenotomy tendon had a normal appearance at 17 weeks. We noted marked variations in the immediate postoperative ultrasound findings, in particular, the level in relation to the calcaneum, the obliquity, and the completeness of the surgical division. Good passive ankle dorsiflexion was achieved in all patients despite ultrasonographically incomplete sectioning of the tendon in 14 of 22 tendons (63 %). The healing process was also variable on ultrasound imaging with several distinct patterns emerging. Functional continuity was noted both clinically and sonographically well before apparent sonographic anatomical continuity of the tendon. For example, passive dorsiflexion and plantarflexion resulted in movement of the proximal and distal tendon in functional continuity despite the presence of an apparent hypoechoic gap between the severed ends. At the initial study, some peripheral tendon fibres were in apparent sonographic continuity in 63 % of cases on either longitudinal or transverse imaging despite an immediate increase of dorsiflexion directly after tenotomy with palpable gap formation. All patients demonstrated fusiform hypoechoic thickening between the tendon ends. It was impossible to distinguish haematoma from amorphous tendon continuity despite the use of high quality apparatus.

**Table 1:** Age at tenotomy and length of ultrasound follow-up in weeks; time between tenotomy and first ultrasound in days

	1	2	3	4	5	6	7	8	9	10	11	12	13	14	15
Age at tenotomy (weeks)	15	36	44	11	13	9	12	9	7	16	17	27	38	10	8
Time from tenotomy to first ultrasound (days)	17	25	12	19	25	27	21	10	17	0	24	17	19	10	24
Ultrasound follow-up (weeks)	52	17	55	37	45	63	55	60	33	45	27	25	26	39	26
Tenotomy side	B	L	B	R	B	R	R	B	L	R	R	B	B	B	L

Clubfoot side: *R* right, *L* left, *B* bilateral

**Table 2:** Width of tenotomy side on longitudinal ultrasound

Patient	US
1	L: FS. R: FS
2	L: N
3	L: N. R: FS
4	R: FS
5	L: FS. R: N
6	R: N
7	R: FS
8	L: N. R: FS
9	L: N
10	R: N
11	R: FS
12	L: FS. R: N
13	L: FS. R: N
14	L: FS. R: N
15	L: FS

*R* right, *L* left, *N* normal, *FS* fusiform swelling

On subsequent early studies (mean 55.2 days after tenotomy, range 45–102 days) irregular hypoechoic tissue persisted in the gap between the severed ends. In 40 % of cases, eight out of 20 indistinct striations appeared within the amorphous hypoechoic bridging tissue. In no cases did the severed ends appear to approximate each other, nor did striations appear centripetally from the paratenon. The apparent gap between the severed ends varied markedly within the same patient depending on the exact scanning plane. This was due to the tendon having been divided obliquely.

In addition, it was possible to produce images of an apparently undivided tendon where the division had been clinically and functionally complete.

**Fig. 1:**
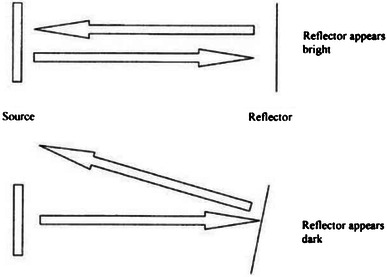
Anisotropy. The tendon (reflector) appears bright when it runs at 90° to the ultrasound beam, but dark when the angle is changed. At smooth boundaries, the angle of reflection and incidence are the same; just as they are with a conventional mirror. However, the probe will only receive the reflected sound if the beam strikes the surface at a right angle

**Fig. 2:**
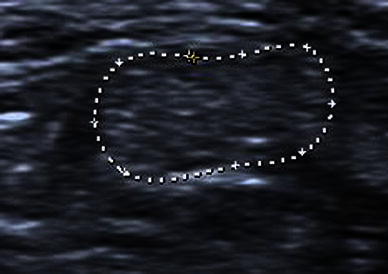
Anisotropy. Transverse image of an intact tendon which shows the characteristic normal fibrillary appearance

**Fig. 3:**
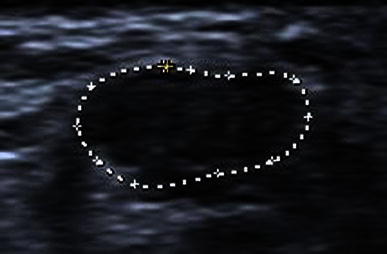
Anisotropy. Transverse image at the identical region of the same tendon as Fig. 2. Here the tendon appears far more hypoechoic. This can be misinterpreted as a defect but it is an ultrasound artefact due to the incident beam not being perpendicular to the tendon

All cases demonstrated the phenomenon of ultrasound anisotropy since this is a universal property of tendon ultrasound. An intact tendon may be misdiagnosed as being discontinuous due to the angle of incidence of the ultrasound beam (Figs. 1, 2, 3).

At the last scan, 12 out of 22 divided tendons showed fusiform swelling at the tenotomy site. Eight divided tendons were scanned at 12–14 months. Five still showed obvious fusiform swelling with amorphous, nonstriated features. The other three looked normal.

### Results for group 2 patients

Nine patients (11 tendons), who had not undergone serial ultrasound monitoring in the immediate postoperative period, were scanned between 34 and 83 months postsurgery (mean 60 months). There were seven patients treated for unilateral CTEV and two treated for bilateral deformity giving 11 tendons that were scanned. Three patients (four tendons: two unilateral and one bilateral) demonstrated entirely normal tendons (33, 47, and 83 months postsurgery). Six patients had abnormal tendons [35, 36, 69, 74, and 83 (×2) months postsurgery]. One patient scanned at 83 months had bilateral surgery with one tendon appearing abnormal and the other normal. The abnormal tendons displayed fusiform swelling with disorganized poorly defined striations, and with an ultrasound appearance, which was unlike the normal controls. The contralateral unaffected Achilles tendon had normal appearances similar to the control group.

### Results for normal controls

We scanned the tendons of seven children with 14 normal feet to have a baseline definition of what is a normal tendon appearance on the ultrasound. All normal controls demonstrated normal echogenic fibrillary pattern in transverse and longitudinal scanning planes with a gradual tapering of the tendon to the tendoachilles insertion and no fusiform swelling. This appearance was similar to a normal contralateral tendon in patients with unilateral clubfeet deformity.

## Discussion

After Graf [11] popularised baby hip ultrasound, ultrasound has been increasingly used to assess orthopaedic abnormalities and the results of orthopaedic treatments in children. Schlesinger et al. [12] and Hamel et al. [13] reported on the use of ultrasound in the assessment of clubfeet and vertical talus. Weigl et al. [14] described sonographic healing stages of Achilles tendons after tenomuscular lengthening (1: haematoma and exudate, 2: organization of haematoma, 3: appearance of connective tissue fibres in the gap, 4: maturation of connective tissue, 5: tendon-like structure, and 6: organization of tendonlike structure) in children with cerebral palsy and stated that the healed tendons were not differentiable from normal tendons. However, the healed tendons were described as fusiform with a markedly increased anteroposterior diameter. Barker and Lavy [8] were the first to visualise the healing of Achilles tenotomies as part of Ponseti clubfoot treatment using ultrasound. They reported that all divided 11 Achilles tendons showed ultrasonographic continuity by six weeks. Mangat et al. [7] reported three phases of healing within the gap zone of a divided Achilles tendon in children with clubfeet with the healing being complete by 12 weeks in the majority of children under the age of 24 months. At six weeks a bulbous mass was still present within the gap. In their patients the transition from regenerating to normal tendon appeared between six and 12 weeks, when the new fibres became indistinguishable from the original tendon. Maranho et al. [6] were the first who measured tendon dimensions ultrasonographically after Achilles tenotomy in clubfeet in 2009. Focal thickening was still present in the repaired area at one year. Foot position and level of scanning at the gap area were not defined. Agarwal et al. [9] measured dimensions in 2012 after Achilles tendon releases in clubfeet. The tendons were scanned first preoperatively with the feet in maximum dorsiflexion at a level approximately 1 cm above the calcaneal insertion. This means that the feet and calcanei will have been in different positions, and the tendons will have been stretched different amounts. The text indicates that the 1 cm distance was estimated giving a further unknown variable increasing the inaccuracy of any measurement. However, the radiologist performing the ultrasounds was able to measure tendon dimensions to a precision of 0.01 mm. The second measurement was performed four weeks after tenotomy, but the foot position and level of the ultrasound were not described at this point. The authors reported that there was no significant difference in Achilles tendon width, thickness, area in normal tendons and in unilateral and bilateral clubfeet before and after tenotomy. However, the provided mean data showed a 28/4/48 % increase of thickness, width and area, respectively, for the normal tendons. A bulbous mass, as previously reported [6, 7] and seen in our study, would have made measurements of thickness, width, and surface area irreproducible since they would change depending on the tendon level scanned and the position of the foot, but Agarwal et al. did not report such a bulbous mass. The mean age at tenotomy was five months in the Agarwal [9] paper and seven weeks in the Maranho et al. [6] paper with a mean thickness of the normal tendons of 1.84 and 2.50 mm, respectively. Therefore, the mean thickness of the latter group was 36 % larger despite the patients mean age being about three times younger.

Tendons are comprised of organised bundles of collagen with a regular, ordered structure. This regular structure results in markedly differing ultrasound properties depending on whether the tendon is scanned in longitudinal or transverse planes. In addition, the ultrasound characteristic will differ between images in similar planes, for example, in transverse planes, as a result of varying angles of the incident ultrasound beam. This property, known as anisotropy, may result in artefactual appearances of pathology, in particular, spurious impressions of rupture or oedema.

Our initial intention was to define the process of tendon healing by serial ultrasound and clinical examinations, including tenotomy gap length and dimensions of the healing tendon. It became apparent that this intention was not feasible due to variability in ultrasound imaging that can be achieved depending on many technical factors that were not mentioned previously in previous studies [6–9]. It is our impression that these studies have oversimplified both the technical challenges as well as the observations.

**Fig. 4:**
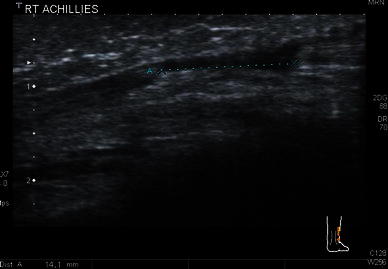
Ultrasound measurement of the tendon gap after Achilles tenotomy with the foot in a neutral position

**Fig. 5:**
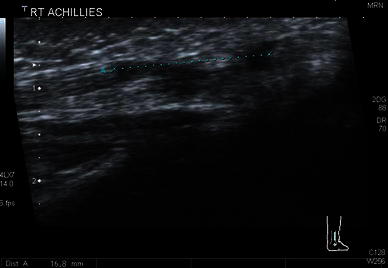
Ultrasound measurement of the tendon gap of the same tendon as Fig. 4 but with the foot in full dorsiflexion. There is a large variation in gap measurement which is greatly affected by the position of the foot

Although measurements have been given for the exact tenotomy gap and tendon widths in static ultrasound images, no mention was made of the position of the foot, which we have found has a large impact on gap measurements [6–9] (Figs. 4, 5). We, therefore, deem that any gap measurements are, in fact, useless.

Our findings are reflected by Niki et al. [10] who reported that numerical ultrasound examination of stumps lacked accuracy because of constant movements and the difficulty of defining the end of the cut tendon stumps based on ultrasound examination in 23 babies with 33 clubfeet even though one observer was versed in ultrasonographic examination. They also reported that there was still a slight irregularity of the internal tendon structure at two years after tenotomy. In a second study Niki et al. [15] investigated calf muscle atrophy after Achilles tenotomies in 36 patients with a unilateral clubfoot measuring transverse and anteroposterior diameters ultrasonographically. They reported that tendon healing and gliding had been achieved 24–34 months after tenotomy. Tendon and calf muscle diameters were found to be significantly smaller on the tenotomy side compared to the unaffected side, but there was no significant difference in changes over time between affected and unaffected sides. The authors pointed out that they did not know if the differences in diameters were the result of the tenotomy or the clubfoot abnormality. These ultrasound measurements were performed despite that they pointed out in their former publication that the ultrasound measurements are inaccurate.

However, we were able to demonstrate varying gaps and even apparent ultrasound continuity in several patients where successful postoperative dorsiflexion was achieved.

We suggest that successful clinical results can be achieved despite variable ultrasonographic appearances and despite an ultrasonographically incomplete tendon division in 63 % of tendons. Ten of our patients (14 feet) were given a Pirani score of 0 directly after tenotomy. The remaining five patients (eight feet) were given a Pirani score [16] of 0 either by the time the first postoperative cast was changed or at the end of the casting period.

In this study an experienced paediatric musculoskeletal ultrasonologist found it impossible to accurately measure gaps between the tenotomized ends because of the dependence on the angle of the probe, the obliquity of the tenotomy, and the variable position of the foot. There is a large variation in gap measurements that can be obtained between the retracted tendon ends. The width measurement is also inaccurate and varies with the position of the probe, which cannot be standardised and may vary between ultrasound studies. This highlights pitfalls in previous papers measuring the distances and producing apparent accurate figures to quantify these gaps.

We were able to visualise the phenomenon of anisotropy. This occurs when the footprint of the ultrasound transducer is not parallel to the tendon and the ultrasound beam is not perpendicular to the long axis of the tendon. This may produce an impression of a tendon defect in a normal continuous tendon, when it is actually intact. The appearances of a normal tendon change a lot depending on the probe position making it also difficult to define the structure of a normal tendon and compare this with the cut tendons.

We agree with other authors that there is a phase of an easily identifiable post-tenotomy bulbous mass consistent with haematoma formation [6].

In our serial studies, we were able to identify striated tissue forming in the gap between the tenotomized tendon ends. This commenced at approximately two months. Despite the appearance of early striations, the bridging tissue remained slightly hypoechoic in contrast to the normal fibrillary structure of the adjacent tendon cephalad and caudal to the tenotomy. In five tendons, even one year after tenotomy, the normal striated structure had not appeared.

**Fig. 6:**
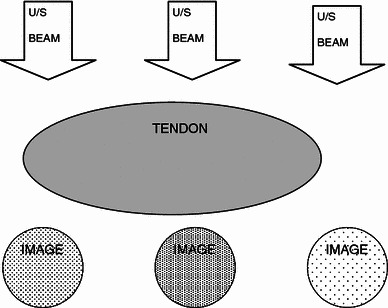
Partial volume artefact—the slice that makes up the ultrasound image isthree-dimensional. This phenomenon is caused by the size of the image voxel. The loss of resolution is caused by multiple features present in the image voxel. In ultrasound imaging that occurs when the slice thickness is the scanned structure

We observed the phenomenon of partial volume effect. Due to finite slice thickness, if a tendon is incompletely tenotomised, a longitudinal image of the intact portion will appear as a normal tendon. A longitudinal image of the adjacent gap will appear as a hypoechoic defect. Both the above are accurate images of the tendon. However, if the longitudinal image includes both the continuous portion and the gap, it will result in an image of hypoechoic (darker) tissue in which there are apparent striations. This may produce a spurious impression of organised fibrillary pattern appearing in hypoechoic healing tissue when the fibrillary-striated pattern is derived from intact adjacent fibres and no healing has actually taken place (Fig. 6).

The majority of previous publications have not included transverse images in their publications [6–8, 12]. Without these, it cannot be concluded that there were no intact peripheral fibres as occurred in several of our cases, despite a clinically successful appearance of dorsiflexion.

In no cases did we observe healing occurring by the extension and approximation of normal fibrillary-patterned tendon ends. In all cases amorphous tissue in the tendon gap develops into apparently a normal tendon.

Despite careful searching, we were unable to demonstrate confidently an intact plantaris in any of the studies.

Previous papers [6–8, 11] give the impression that the Achilles tendon after percutaneous tenotomy in children heal with a normal structure and appearance on ultrasound albeit with increased diameter. Our study shows that the ultrasonographic appearances in the vast majority of patients do not normalise in our group 1 patients and also remain abnormal in some patients several years after tenotomy. Although the Ponseti technique has been undertaken for over 30 years, we are unaware of reports of an increased risk of longer term consequences such as the development of Achilles tendon degeneration and rupture in patients who had an Achilles tenotomy as part of their treatment, and we do not know what the divided tendons are like ultrasonographically in adulthood.

## Conclusion

There is a previously undescribed stage of normal functioning tendon that cannot be demonstrated by static ultrasound images. Routine ultrasound studies are of no value as an adjunct to clinical assessment either intraoperatively or for postoperative monitoring, as the desired postoperative result of sufficient passive dorsiflexion of the foot is independent of the ultrasound impression of the completeness, obliquity and level of the tenotomy. Furthermore, the images may give misleading information regarding the need to complete the tenotomy, which may increase risks associated with a further pass of the scalpel blade as well as increasing the length of the surgical procedure as has been suggested in a previous study [6].
